# *β*-Hydroxy-*β*-Methylbutyrate Free Acid Attenuates Oxidative Stress Induced by a Single Bout of Plyometric Exercise

**DOI:** 10.3389/fphys.2019.00776

**Published:** 2019-06-25

**Authors:** Hamid Arazi, Zahra Hosseini, Abbas Asadi, Rodrigo Ramirez-Campillo, Katsuhiko Suzuki

**Affiliations:** ^1^Department of Exercise Physiology, Faculty of Sport Sciences, University of Guilan, Rasht, Iran; ^2^Bandar Anzali Branch, Islamic Azad University, Bandar Anzali, Iran; ^3^Department of Physical Education and Sport Sciences, Payame Noor University, Tehran, Iran; ^4^Laboratory of Human Performance, Quality of Life and Wellness Research Group, Department of Physical Activity Sciences, Universidad de Los Lagos, Osorno, Chile; ^5^Faculty of Sport Sciences, Waseda University, Tokorozawa, Japan

**Keywords:** leucine, plyometric training, free radicals, oxidative stress, stretch-shortening cycle

## Abstract

**Purpose:**

The purpose of this study was to examine the effect of β-hydroxy-β methylbutyrate free acid (HMB-FA) ingestion on oxidative stress and leukocyte responses to plyometric exercise.

**Methods:**

In a randomized double-blind placebo-controlled design, physically active males were assigned to the HMB-FA (*n* = 8) or placebo (*n* = 8) groups that consumed either 1 g of HMB-FA or placebo 30 min prior to performing an acute plyometric exercise protocol (15 sets of 10 repetitions of maximal-effort vertical jumps). Blood was obtained pre-(T1), post-(T2), and 1-h post-(T3) exercise to determine changes in serum levels of 8-hydroxy-2-deoxyguanosine (8-OHdG), malondialdehyde (MDA), protein carbonyl (PC), and white blood cells (WBC).

**Results:**

The exercise protocol significantly elevated 8-OHdG (HMB-FA, T2 9.5 and T3 12.6%; placebo, T2 18.2 and T3 36.5%), MDA (HMB-FA, T2 11.6 and T3 25.2%; placebo, T2 11.8 and T3 41%) and PC (HMB-FA, T2 6.9 and T3 25%; placebo, T2 23.4 and T3 55.3%) at post- and 1-h post-exercise, respectively. However, at 1-h post-exercise, greater increases in oxidative stress markers (8-OHdG 36.5 vs. 12.6%; MDA 41 vs. 25.1% and PC 55.3 vs. 25%) were observed in the placebo group compared to the HMB-FA group (*p* < 0.05). In addition, the WBC level was greater for the placebo group in comparison to the HMB-FA group at post-exercise.

**Conclusion:**

HMB-FA attenuated oxidative stress and leukocyte responses to plyometric exercise compared with placebo.

## Introduction

Exercise could increase oxygen utilization 200-fold above resting level in active muscles resulting in oxidative stress (Bloomer et al., 2005). Exercise-induced oxidative stress is associated with increasing free radicals and reactive oxygen species (ROS) (Bloomer et al., 2005). In fact, intense exercise (e.g., resistance and/or eccentric exercise) has been shown to increase the circulation of neutrophils, elastase and myeloperoxidase, resulting in free radical production, oxidative stress, and inflammation (Rahimi, 2011; Suzuki, 2017).

Some experimental human models have addressed the associations between aerobic and anaerobic exercise-induced oxidative stress and its acute effects on oxidative damage (Bloomer et al., 2005; Rahimi, 2011). However, to the best of our knowledge, no human model has addressed the effect of plyometric exercise on these variables. Although plyometric training is extensively used to improve jumping and power-based physical fitness variables (Asadi et al., 2017), an acute session of plyometric training may induce muscle damage and inflammation responses in humans (Twist and Eston, 2007; Arazi et al., 2016). For instance, Chatzinikolaou et al. (2010) investigated changes in inflammation and leukocyte responses after acute plyometric exercise in men and found increases in leukocyte count, creatine kinase and lactate dehydrogenase activities, C-reactive protein, and interleukine 6 (IL-6) at immediately post- to 24-h post-exercise. In fact, it seems that to prevent muscle damage, inflammation and maybe oxidative stress, increasing the dietary content of nutritional antioxidants could play a crucial role to manage these responses (Street et al., 2011).

It appears that supplementation with vitamins, proteins, mixed amino acids, and branched-chain amino acids may be effective in reducing the leukocyte response and attenuating inflammation following eccentric exercise (Calder, 2006; Bobeuf et al., 2011; Atashak et al., 2014). β-hydroxy-β-methylbutyrate (HMB) is a metabolite of the amino acid leucine, a potent stimulus of translation initiation and protein synthesis (Fitschen et al., 2013). Previous studies have reported that HMB supplementation could reduce symptoms and indirect markers of muscle damage such as soreness and creatine kinase activity following exercise (Hoffman et al., 2004; Wilson et al., 2009). In addition, some studies reported that HMB supplementation induces antioxidant effects by improving immune function, increasing the number of CD3+CD8+ cells, and attenuating circulating levels of pro-inflammatory cytokines (Nissen and Abumrad, 1997; Peterson et al., 1999; Clark et al., 2000; Hsieh et al., 2006).

To date, a large number of studies examined the effects of calcium salt form of HMB (HMB-Ca) on inflammation and muscle damage. However, recently it appears that the free acid form of HMB (HMB-FA) has greater bioavailability and plasma concentrations in a shorter time when compared with the HMB-Ca (36 vs. 131 min) (Fuller, Sharp et al., 2011). In fact, Fuller, Sharp et al. (2011) observed that HMB-FA results in two-fold greater plasma HMB levels compared to HMB-Ca, and the greater concentration level was achieved four times faster, with 25% greater clearance by the body, indicating improved utilization.

Recent studies showed that HMB-FA supplementation attenuates circulating inflammatory cytokines, muscle damage and muscle soreness after exercise, with antioxidant effects *in vivo* (Nissen and Abumrad, 1997; Peterson et al., 1999; Hsieh et al., 2006; Gonzalez et al., 2014; Arazi et al., 2018). However, no information is available regarding the effects of HMB-FA ingestion on plyometric exercise-induced oxidative stress in humans. Additionally, the effect of HMB on exercise-induced oxidative stress is unknown. Therefore, the aim of this study was to examine the efficacy of HMB-FA as a nutritional intervention on oxidative stress and leukocytes after plyometric exercise. We hypothesized that HMB-FA ingestion at pre-exercise would reduce oxidative stress and leukocyte responses in humans.

## Materials and Methods

### Participants

Sixteen healthy college-aged men (21.6 ± 1.2 year) participated in this study. The participants were familiar with plyometric exercise/training. The participants were randomly assigned in a double-blind manner to a HMB-FA (*n* = 8) or placebo (*n* = 8) groups (Table 1). A sample size of eight participants per treatment produced a statistical power (1-β) of 0.97. Power calculations were made using G^∗^Power statistical analyses software (Version 3.1.9.2, Düsseldorf, Germany) and were based upon an effect size (dz) of 0.91 generated from changes in muscle damage indices reported by Twist and Eston (2007) in response to lower body plyometric exercise. Participants were free from any lower body injuries and had not used any supplement within the past 6 months prior to inclusion in this study. The participants were instructed to avoid any physical exercise for at least 7 days prior to experiments. Participants were informed about the nature of the study protocol and the associated risks and benefits. This study was approved by the Ethics Research Committee of the University of Guilan (DT/2248/2017) and IAU Bandar-Anzali Branch (No. 1374/2017) and the participants read and signed the information in consent form that was in accordance with the Declaration of Helsinki.

**Table 1:** Participants’ characteristics.

	HMB-FA (*n* = 8)	Placebo (*n* = 8)
Age (y)	22 ± 1	21.5 ± 1.1
Height (cm)	183.3 ± 6.6	181.3 ± 6.3
Weight (kg)	81.5 ± 5.3	79.2 ± 5.8
Training experience (y)	4.2 ± 2.8	4.6 ± 2.4
Maximal vertical jump (cm)	41.7 ± 5.3	41.8 ± 4.8

*HMB-FA, β-hydroxy-β-methylbutyrate-free acid. Data are presented as mean ± SD.*

### Experimental Protocol

This study utilized a placebo-controlled, double-blind, simple randomized design. Participants reported to the laboratory on two occasions. On the first visit, participants’ height (Seca 222, Terre Haute, IN, United States) and body mass (Tanita, BC-418MA, Tokyo, Japan) were measured, and participants were tested for their maximal vertical jump as described previously (Arazi et al., 2016). During this session, the participants were familiarized with proper technique of plyometric exercise and were instructed to achieve 90° of knee flexion followed by 5 jumps for maximum height. 1 week after the first visit, the participants reported to the laboratory again and completed a session of plyometric exercise, which consisted of 15 sets of 10 repetitions of maximal-effort vertical jumps. The participants ingested 1 g of HMB-FA or placebo (i.e., polydextrose) 30 min prior to exercise, and blood samples were drawn at pre-(T1), post-(T2) and 1-h post-(T3) exercise to analyze changes in serum oxidative stress markers [8-hydroxy-2-deoxyguanosine (8-OHdG), malondialdehyde (MDA), and protein carbonyl (PC)], and leukocytes [white blood cells (WBC), neutrophils, lymphocytes, and monocytes].

### HMB-FA Ingestion

The participants ingested 1 g of HMB-FA (free acid gel, Body Attack, GmbH & Co., KG, Waldhofstrabe 19, 25474, Ellerbek, Germany) or placebo (polydextrose) 30 min prior to the exercise. The supplement and placebo were similar in appearance and taste, and ingestion was witnessed by one of the study investigators (Townsend et al., 2013; Gonzalez et al., 2014).

### Diet Control

To control potentially confounding effects of diet, the participants completed a diet recall for 3 days before their inclusion in the study (Wilson et al., 2014). Records were analyzed by Nutritionist IV diet analysis software for total calories, protein, carbohydrate, fat, vitamin C, and vitamin E intake (Table 2).

**Table 2:** Dietary intake assessed during the 3 days before exercise session.

	Energy intake (kcal)	Carbohydrate (g)	Fat (g)	Protein (g)	Vitamin E (mg)	Vitamin C (mg)
HMB-FA	2431 ± 320	250 ± 14	73 ± 15	95 ± 31	8.8 ± 1.2	70 ± 25
Placebo	2467 ± 279	255 ± 21	70 ± 17	93 ± 29	8.6 ± 1.9	72 ± 32

*HMB-FA, β-hydroxy-β-methylbutyrate-free acid. Data are presented as mean ± SD.*

### Plyometric Exercise

After 10 min of warm-up (i.e., 5 min cycling and 5 min stretching movements), the participants performed 15 sets of 10 maximal-effort vertical countermovement jumps, with 1 min of rest between sets. The participants were instructed to perform each jump with maximal effort to reach the maximal height. During landing, the participants were instructed to flex the knee joint to ∼90°. The participants received verbal encouragement throughout the exercise session. An experienced strength and conditioning coach monitored all exercise protocols.

### Blood Sampling and Analysis

Blood samples were drawn from each participant at pre, post- and 1 h post-exercise session. Participants were asked to visit the laboratory following a 12-h fast and 8-h of sleep (i.e., 9–11 A.M), which was confirmed by a personal interview before measurements were done. A 5-mL blood sample was collected by venipuncture of an antecubital vein; the resultant samples were allowed to clot at room temperature for 30 min and then centrifuged at 1500 × *g* for 10 min. The serum was then pipetted into polyethylene tubes and frozen at −80°C for subsequent analysis. The serum 8-OHdG, MDA and PC levels were measured using commercially available enzyme-linked immunosorbent assays (ELISA) kits (ZellBio GmbH Veltlinerweg 29, 89075, Ulm, Germany). Complete blood cell analyses were conducted using automated hematology analyzer (PocH100i, Sysmex, Kobe, Japan). The analysis included counting of WBC, neutrophils, lymphocytes, and monocytes. Pre-(T1) blood samples were drawn following a 15 min equilibration period prior to exercise. Post-(T2) blood samples were taken within 5 min of exercise cessation. Following the plyometric exercise protocol, participants remained in the seated position for the full 1-h recovery phase prior to the 1-h post-(T3) blood samples being drawn. The coefficient of variation for all blood measurements was <7%.

### Statistical Analyses

Prior to statistical comparison, all data were checked for normal distribution by the Shapiro–Wilk test. Data were presented as mean ± standard deviation (SD) and analyzed by 2-way [2 (group) × 3 (time)] repeated-measures analysis of variance (ANOVA) using the SPSS statistical software package (SPSS version 16.0 for Windows, SPSS Inc., Chicago, IL, United States). When a significant F value was achieved, a Bonferroni *post hoc* test was used to detect differences in the measures. Assumptions of sphericity were assessed using Mauchly’s test of sphericity, with any violations adjusted by use of the Greenhouse-Geisser (GG) correction. In the event of a significant interaction, follow up one-way ANOVA’s were used to compare changes across 3 time points (T1, T2 and T3). The area under the curve (AUC) for all hormone concentrations was calculated by using a standard trapezoidal technique and was analyzed using an independent *t*-test across groups. Independent-sample *t*-tests were also performed to determine possible group differences for the following variables: height, body mass and dietary intake. Effect sizes (ES) were calculated using Cohen’s *d*. Threshold values for assessing magnitudes of ES were 0.20, 0.60, 1.2, and 2.0 for small, moderate, large, and very large, respectively (Hopkins et al., 2009). Moreover, for a percent change score differences between pre and all subsequent time points were calculated. An alpha level of *p* ≤ 0.05 was accepted as a statistically significant difference between experimental conditions.

## Results

At baseline, no significant differences were observed between groups for height, body mass, diet records or other variables (i.e., 8-OHdG, MDA, PC, WBC, neutrophils, lymphocytes, and monocytes) (*p* > 0.05).

**FIGURE 1:**
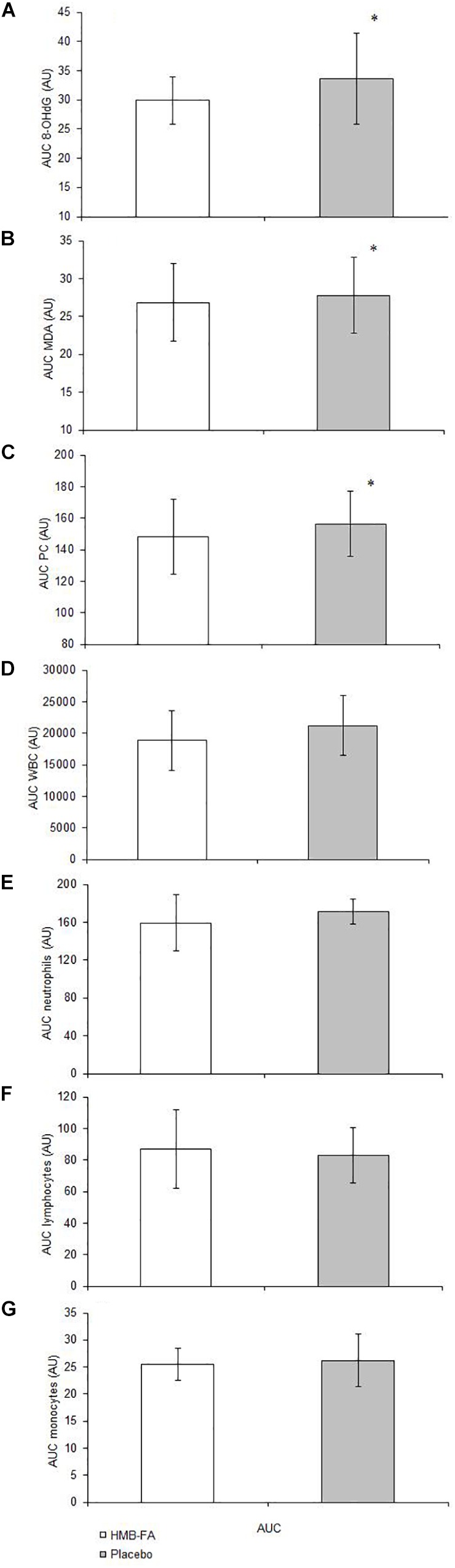
Area under the curve (AUC) analysis for dependent variables in the HMB-FA and placebo groups. **(A)** 8-hydroxy-2-deoxyguanosine (8-OHdG), **(B)** malondialdehyde (MDA), **(C)** protein carbonyl (PC), **(D)** white blood cells (WBC), **(E)** Neutrophils, **(F)** Lymphocytes, and **(G)** Monocytes. Data were reported as mean ± SD. ^∗^Significant (*p* ≤ 0.05) difference between groups.

There was a significant group × time interaction for 8-OHdG [*F*_(1.4,20.7)GG_ = 3.7, *p* = 0.03]. Both the HMB-FA and placebo groups showed increases in serum 8-OHdG from T1 to T2 (*p* = 0.02) and T3 (*p* = 0.01), however, serum 8-OHdG was significantly greater in the placebo at T3 compared to HMB-FA (*p* = 0.001). The AUC analysis for 8-OHdG (Figure 1A) revealed greater increases for the placebo compared to HMB-FA (*p* = 0.04).

There was a significant group × time interaction for MDA [*F*_(1.4,20.7)GG_ = 2.8, *p* = 0.021]. Both the HMB-FA and placebo groups showed increases in serum MDA from T1 to T2 (*p* = 0.04) and T3 (*p* = 0.023), however, serum MDA was significantly greater in the placebo at T3 compared to HMB-FA (*p* = 0.001). The AUC analysis for MDA (Figure 1B) revealed greater increases for the placebo compared to HMB-FA (*p* = 0.044).

There was a significant group × time interaction for PC [*F*_(1.4,20.7)GG_ = 3.4, *p* = 0.012]. Both the HMB-FA and placebo groups showed increases in serum PC from T1 to T2 (*p* = 0.05) and T3 (*p* = 0.001), however, serum PC was significantly greater in the placebo at T3 compared to HMB-FA (*p* = 0.001). The AUC analysis for PC (Figure 1C) revealed greater increases for the placebo compared to HMB-FA (*p* = 0.021).

There was a significant group × time interaction for WBC [*F*_(1.3,18.6)GG_ = 13.8, *p* = 0.044]. Both the HMB-FA and placebo groups showed increases in WBC from T1 to T2 (*p* = 0.011), however, WBC was significantly greater in the placebo at T2 compared to HMB-FA (*p* = 0.001). The AUC analysis for WBC (Figure 1D) revealed no significant differences (*p* = 0.638) between the groups.

There was no significant group × time interaction for neutrophils [*F*_(1.4,20.7)GG_ = 2.8, *p* = 0.6]. Both the HMB-FA and placebo groups showed increases in neutrophils from T1 to T3 (*p* = 0.001). The AUC analysis for neutrophils (Figure 1E) revealed no significant differences (*p* = 0.512) between the groups.

There was a significant group × time interaction for lymphocytes [*F*_(1.8,25.6)GG_ = 3.5, *p* = 0.38]. The placebo group showed increases in lymphocytes from T1 to T2 (*p* = 0.008), and lymphocytes were significantly greater in the placebo at T2 compared to HMB-FA (*p* = 0.021). No significant changes were seen at any time point for the HMB-FA group in response to plyometric exercise (*p* > 0.05). The AUC analysis for lymphocytes (Figure 1F) revealed no significant differences (*p* = 0.481) between the groups.

There was no significant group × time interaction for monocytes [*F*_(1.4,20.3)GG_ = 0.39, *p* = 0.23]. Both the HMB-FA and placebo groups showed increases in monocytes from T1 to T2 (*p* = 0.03), and T3 (*p* = 0.01). The AUC analysis for monocytes (Figure 1G) revealed no significant differences (*p* = 0.262) between the groups.

## Discussion

Several studies have reported that physical exercise results in elevations in ROS resulting in oxidative stress (Suzuki et al., 1996; Bloomer et al., 2005; Bloomer et al., 2006; Rahimi, 2011), whereas antioxidant supplementation could reduce the symptoms of oxidative stress and inflammation after exercise (Peake and Suzuki, 2004; Peake et al., 2007). To the best of our knowledge, the present study is the first to examine the effects of HMB-FA ingestion prior to plyometric exercise on oxidative stress and inflammatory response in humans. In this study, plyometric exercise resulted in elevations of 8-OHdG, MDA, and PC after exercise, with greater elevation for the placebo group compared to the HMB-FA group.

In this study we found increases in 8-OHdG at T2 and T3 (see Table 3) which is in accordance to previous studies which have reported an increase in DNA damage after eccentric exercise (Bloomer et al., 2005; Calder, 2006; Rahimi, 2011; Atashak et al., 2014). The possible mechanisms for this situation could be due to anoxic state after eccentric (i.e., plyometric) exercise in the exercising muscle (Uchiyama et al., 2006), activation of neutrophils (Suzuki et al., 1996; Peake and Suzuki, 2004; Suzuki, 2017), or xanthine oxidase, a source of superoxide anions during eccentric exercise (Margonis et al., 2007). In relation to HMB, most studies used the calcium form of HMB and found conflicting results (Hoffman et al., 2004; Wilson et al., 2009). In this study the participants consumed HMB-FA, which has been shown to result in greater availability and absorbance compared to calcium form of HMB (Fuller, Sharp et al., 2011), which may induce antioxidant effects by improving immune function, and attenuating circulating levels of pro-inflammatory cytokines (Nissen and Abumrad, 1997; Peterson et al., 1999; Clark et al., 2000). In fact, ingestion of HMB-FA prior to exercise induces anti-catabolic and protective effects to reduce oxidative stress (Hsieh et al., 2006; Arazi et al., 2018), which are in accordance with our results, indicating that ingestion of HMB-FA prior to plyometric exercise attenuated the rise in serum 8-OHdG levels at T3 compared with the placebo group.

In line with our finding on the increase of serum MDA after exercise, it appears that eccentric exercise induced lipid peroxidation (i.e., MDA); however, to date, some studies have reported elevation (Rahimi, 2011) and no changes (Radak et al., 1999; Ramel et al., 2004) in MDA post-exercise. In fact, polyunsaturated fatty acids makes cellular membranes and eccentric exercise-induced muscle damage increases blood concentration of polyunsaturated fatty acids resulting in lipid peroxidation (i.e., MDA) (Nikolaidis et al., 2008). Regarding reports from previous studies, nutritional supplementation could play an important role to attenuate the rise in serum MDA levels (Nikolaidis et al., 2008; Rahimi, 2011). In this study, HMB-FA ingestion prior to plyometric exercise attenuated the rise in serum MDA levels at T3 compared with the placebo group. It seems that the Cholesterol Synthesis Hypothesis (CSH) may have occurred. According to the CSH, muscle cell damage after eccentric exercise may exceed the capacity of cells to produce adequate amounts of cholesterols to maintain sarcolemmal integrity, which is important for muscle tissue. Cholesterol is formed from Acetyl-CoA catalyzed by the enzyme HMG-CoA reductase, and most HMB is converted into HMG-CoA reductase (Albert et al., 2015). Therefore, it seems that HMB-FA ingestion prior to plyometric exercise induced increases in intramuscular HMB concentrations and substrate for the synthesis of cholesterol to maintain sarcolemma integrity and inhibit cell membrane breakdown, limiting MDA production in the blood (Albert et al., 2015).

**Table 3:** Changes in variables for the HMB-FA and placebo groups following plyometric exercise.

		Time point values			T1 to T2		T1 to T3		T2 to T3	
		T1	T2	T3	ES (95% CI)	% of change	ES (95% CI)	% of change	ES (95% CI)	% of change
8-OHdG (ng/mL)	HMB-FA	9.28 ± 1.8	10.16 ± 0.9^∗^	10.45 ± 1.4^∗^	0.62 (−0.42 to 1.59)	9.5	0.82 (−0.24 to 1.79)	12.6	0.25 (−0.75 to 1.22)	2.9
	Placebo	9.46 ± 2.2	11.18 ± 2.6^∗^	12.91 ± 3.1^∗†‡^	0.76 (−0.33 to 1.68)	18.2	1.28 (−0.15 to 2.28)	36.5	0.64 (−0.39 to −1.61)	15.5
MDA (nmol/mL)	HMB-FA	7.97 ± 2	8.9 ± 1.6^∗^	9.98 ± 1.5^∗^	0.51 (−0.51 to 1.48)	11.6	1.14 (−0.03 to 2.12)	25.2	0.7 (−0.35 to 1.66)	12.1
	Placebo	7.87 ± 1.9	8.8 ± 2.1^∗^	11.1 ± 1^∗†‡^	0.46 (−0.55 to 1.43)	11.8	2.13 (0.81 to 3.21)	17.9	1.4 (0.24 to 2.4)	26.1
PC (ng/mL)	HMB-FA	45.17 ± 4.1	48.2 ± 10.2^∗^	56.5 ± 7^∗^	0.4 (−0.61 to 1.37)	6.8	1.98 (0.68 to 3.04)	25	0.94 (−0.14 to 1.92)	17
	Placebo	41.4 ± 6	51.1 ± 6.3^∗^	64.3 ± 7.3^∗†‡^	1.58 (0.38 to 2.6)	23.4	3.43 (1.75 to 4.73)	55	1.94 (0.66 to 3)	25.8
WBC (cells/μL)	HMB-FA	5895 ± 1580	7082 ± 1692^∗^	5900 ± 1476	0.73 (−0.32 to 1.69)	20.8	0.01 (−0.96 to 0.98)	0.1	−0.74 (−1.71 to 0.31)	−17
	Placebo	5782 ± 1411	9135 ± 1464^∗‡^	6265 ± 1750	2.23 (0.96 to 3.45)	58	0.3 (−0.7 to 1.27)	8.3	−1.78 (−0.54 to −2.8)	−10
Neutrophils (%)	HMB-FA	54.6 ± 9.2	55@ 10.5	59.1 ± 9.4^∗^	0.04 (−0.94 to 1.02)	0.7	0.48 (−0.54 to 1.45)	12.7	0.41 (−0.6 to 1.38)	7.5
	Placebo	54.8 ± 2.1	56.1 ± 5.2	61.8 ± 5.5^∗^	0.33 (−0.68 to 1.3)	2.4	1.68 (0.47 to 2.71)	12.7	1.07 (−0.03 to 2.05)	10.1
Lymphocytes (%)	HMB-FA	29.5 ± 7.7	29.8 ± 9.5	26.8 ± 7.3	0.03 (−0.95 to 1.01)	1	−0.36 (−1.33 to 0.65)	−9.1	−0.35 (−1.32 to 0.65)	−10
	Placebo	29.7 ± 4.5	34.2 ± 7.1^∗‡^	26.6 ± 5.1	0.76 (−0.3 to 1.73)	15.1	−0.64 (−1.61 to 0.39)	−10.4	−1.23 (−2.22 to 0.1)	−22
Monocytes (%)	HMB-FA	7.5 ± 0.5	8.91 ± 1.1^∗^	9.1 ± 1.5^∗^	1.65 (0.44 to 2.68)	18.8	1.43 (0.27 to 2.44)	21.3	0.14 (−0.84 to 1.12)	2.3
	Placebo	7.6 ± 1.7	9.1 ± 1.6^∗^	9.9 ± 1.8^∗^	0.91 (−0.17 to 1.88)	19.7	1.33 (−0.17 to 2.31)	30.2	0.47 (−0.55 to 1.44)	8.8

*^∗^Denotes differences compared to T1, p ≤ 0.05, ^†^denotes differences compared to T2, p ≤ 0.05, ^‡^denotes differences compared to HMB-FA, p ≤ 0.05. 8-OHdG, 8-hydroxy-2-deoxyguanosine; MDA, malondialdehyde; PC, protein carbonyl; WBC, white blood cells; T1, Pre; T2, Post; T3, 1-h post. Values are mean ± SD. Effect size, ES [95% confidence interval (CI)].*

In line with our findings on the increase in serum PC after plyometric exercise, previous studies have reported that eccentric exercise induces increases in carbonylation of proteins (Margonis et al., 2007; Atashak et al., 2014). The concentration of PC in the blood could be in line with elevation of muscle damage induced by eccentric exercise and proteasome pathways. Ingestion of dietary antioxidants could play an important role in increasing the ability of antioxidant systems (Street et al., 2011). Previous studies have reported that HMB has antioxidant effects to reduce and manage muscle damage and inflammation after exercise (Clark et al., 2000; Hsieh et al., 2006; Arazi et al., 2018). In fact, ingestion of HMB-FA 30 min prior to eccentric exercise blunted the secretion of PC at T3 when compared with the placebo group. This finding is consistent with the mechanisms proposed by Eley et al. (2007), in which HMB attenuates muscle-protein breakdown and its signaling for production of ROS. It seems that HMB is unique in its capacity to increase protein synthesis and anabolic signaling while simultaneously decreasing proteolytic events. This suggests that HMB may inhibit intracellular protein degradation and decrease ubiquitin-proteasome proteolysis pathway (Albert et al., 2015), resulting in PC decrement.

In WBC, the HMB-FA and placebo groups indicated increases at T2, and the placebo groups showed greater significant increases when compared to HMB-FA. Both groups showed similar increases in neutrophils and monocytes after exercise. However, the placebo group indicated significantly greater increases in lymphocytes than the HMB-FA group at T2. The lack of data regarding effects of HMB-FA ingestion prior to plyometric exercise on the leukocyte response makes it difficult to compare with previous findings. However, our results are similar to those previously reported after resistance (Pizza et al., 1996) and plyometric exercises (Doussett et al., 2007; Chatzinikolaou et al., 2010). In our study, we found that HMB-FA ingestion prior to plyometric exercise has protective effects, blunting the WBC and lymphocyte responses to exercise. The prevention of increased WBC and lymphocytes after eccentric exercise could be due to upregulation of antioxidant mechanisms and anti-inflammatory cytokines following contractions by HMB-FA ingestion, and decrements in expression of pro-oxidant enzymes (Suzuki et al., 2003; Peake et al., 2007; Azizbeigi et al., 2013; Atashak et al., 2014). These findings revealed that ingestion of 1 g HMB-FA prior to exercise induced decreases in oxidative stress and leukocytes, and possible mechanism of HMB action is attenuating the inflammatory response to eccentric exercise; however, further studies are necessary to determine the effects of HMB-FA supplementation on inflammation and other mechanisms of action after exercise.

## Conclusion

Our findings provide initial support for the efficacy of HMB-FA supplementation in promoting recovery from eccentric exercise-induced oxidative stress. Findings reveal a potential blunted or delayed oxidative stress and leukocyte responses following plyometric type eccentric exercise with HMB-FA supplementation. Acute HMB-FA supplementation attenuated the magnitude of increases in oxidative stress and leukocytes in comparison with placebo. These findings provide essential information in an attempt to fully understand the effect of plyometric-type eccentric exercise on leukocyte responses and add to our knowledge of mechanisms of oxidative stress response to eccentric exercise. Further researches are needed to investigate the influence of HMB-FA on post-eccentric exercise and to confirm the efficacy of HMB-FA supplementation in improving recovery from exercise-induced oxidative stress and damage.

## Ethics Statement

This study was carried out in accordance with the recommendations of the “Ethics Research Committee of the University of Guilan and IAU Bandar-Anzali Branch” with written informed consent from all subjects. All subjects gave written informed consent in accordance with the Declaration of Helsinki. The protocol was approved by the “Ethics Research Committee of the University of Guilan” (DT/2248/2017) and the IAU Bandar-Anzali Branch (No. 1374/2017).

## Author Contributions

HA designed the study, collected the data analyses, and wrote the manuscript. ZH and AA were involved in acquisition, analysis, and interpretation of data, and reviewed and edited the manuscript. RR-C and KS made a substantial and intellectual contribution to the conception and a critical revision of the manuscript. All authors read and approved the final version of the manuscript.

## Conflict of Interest Statement

The authors declare that the research was conducted in the absence of any commercial or financial relationships that could be construed as a potential conflict of interest.
